# Sustained Suppression of Premature Ventricular Contractions by a Three-Month Pacing Adjustment

**DOI:** 10.7759/cureus.31209

**Published:** 2022-11-07

**Authors:** Henry Sackin, David Campbell, Julie Werth, Jose Nazari

**Affiliations:** 1 Physiology, Rosalind Franklin University of Medicine and Science, North Chicago, USA; 2 Cardiology, NorthShore University Health System, Highland Park, USA; 3 Cardiac Electrophysiology, NorthShore University Health System, Highland Park, USA

**Keywords:** noninvasive, resynchronization, pacemaker, pacing, ablation, cardiomyopathy, ectopic beats

## Abstract

Premature ventricular contractions (PVCs) that comprise more than 15% of total heartbeats can induce cardiomyopathy in patients with systolic dysfunction, and cardiac ablation is frequently used to reduce PVCs in this patient group. However, cardiac ablation is not entirely without hazards. We report a noninvasive method that dramatically reduced PVCs in a cardiac pacemaker patient from 31% to 3% in seven days by increasing the lower limit pacing rate from 50 beats per minute (bpm) to 60 bpm. Not only were our patient's PVCs reduced by the initial pacing elevation, but PVC levels were maintained below 5% even after the pacemaker's lower limit was returned to its original value of 50 bpm. This irreversible suppression of PVC activity following a three-month pacing elevation is a novel result that might be caused by ventricular remodeling of the original ectopic focus.

## Introduction

Premature ventricular complexes (PVCs) are extrasystoles resulting from early depolarizations of the ventricular myocardium. Although PVCs occur in a broad spectrum of the population they differ in normal patients vs. those with underlying myocardial disease. A number of mechanisms have been proposed to explain PVC origin: (1) reentry, after a prior infarction that facilitates conduction delay, (2) abnormal automaticity, associated with electrolyte disturbances, or (3) triggered activity, resulting from afterdepolarizations that reach threshold potential.

PVC burdens of less than 10% of total beats are usually considered benign in patients having no evidence of structural heart disease, whereas PVC burdens above 24% may eventually cause cardiomyopathy [1]. However, for patients with compromised cardiac function, even a PVC burden as low as 10% can eventually lead to cardiomyopathy and a reduction in left ventricular ejection fraction [1-6]. Furthermore, suppression of PVC activity significantly improves cardiac resynchronization therapy (CRT) in patients with left bundle branch block (LBBB). However, the decision to use cardiac ablation on patients with PVC levels in the neighborhood of 10% to 24% involves a risk-benefit analysis since ablation itself is not without hazard. In a study of 1230 patients treated for PVC burden, the overall ablation complication rate was 2.7%, with major adverse cardiac events occurring in 1.5% of patients [7]. Ectopic location (left ventricle and epicardium) was the main predictor of complications, while loci in the right ventricular outflow tract (RVOT) resulted in many fewer problems with ablation [7].

The present case report describes a noninvasive pacemaker protocol for reducing PVCs in a CRT patient whose left ventricle activity was remotely monitored for 11 months. Part of the treatment regimen also consisted of periodic adjustments in the patient's pacing algorithm to investigate the effect and reversibility of lower limit rate changes on PVC activity.

## Case presentation

A physically active 74-year-old man was diagnosed with New York Heart Association (NYHA) class II heart failure, based on reported symptoms of exertional dyspnea, LBBB, limited left ventricular wall motion, and a reduced ejection fraction of 34%, as determined by transthoracic echocardiography (TTE; 2D, M-mode, color and spectral doppler with contrast). Heart failure was judged to be the long-term result of a previous myocardial infarction (MI) that occurred 20 years earlier and produced structural damage to the apex and mid-apical anterior septum. More recent development of LBBB also contributed to the heart failure and low ejection fraction. Since guideline-directed medical therapy was unable to raise ejection fraction, we made the decision to treat the LBBB with a biventricular Medtronic CRT-D pacemaker (Medtronic, Minneapolis, MN, USA) to improve cardiac synchrony. Within a week, the Medtronic CRT-D with DDDR 50 pacing mode elevated the patient's ejection fraction from 34% to 37% as measured by TTE.

Medical therapy for the patient's heart failure and hypertension consisted of ramipril (10 mg q.d.), nebivolol (10 mg q.d.), and eplerenone (25 mg q.d.). Lipid reduction was achieved with evolocumab (140 mg autoinjected every two weeks) and rosuvastatin (5 mg q.d.); and cardiac risk reduction was accomplished with clopidogrel (75 mg q.d.) and baby aspirin (81 mg q.d.). Blood work indicated electrolytes and blood pressure (132/80) within normal limits. Lipids were below normal, with total cholesterol of 87 mg/dL, triglycerides 108 mg/dL, high-density lipoprotein (HDL) 47mg/dL, and low-density lipoprotein (LDL) 18mg/dL. Mild mitral regurgitation and moderate enlargement of the left ventricle were noted.

Since the patient's normal sinus rhythm was a reliable initiator of cardiac contraction, the primary function of the Medtronic CRT was not to suppress the patient's own beat but simply to synchronize contraction of left and right ventricles. Consequently, the pacemaker algorithm was initially set to DDDR 50, because the lower limit setting of 50 beats per minute (bpm) would coincide with the patient's baseline sinus rhythm of 49 ± 2 bpm that was a consequence of daily dosing with nebivolol (10 mg). If heart rate (HR) fell below 50 bpm, the algorithm would adjust HR upward to 50 bpm. Throughout the year-long period covered by this report (1-13-21 to 12-9-21) lower limit settings were never automatically adjusted for patient physical activity. Finally, right ventricle contractions were monitored but electrical stimulation of the right ventricle was generally not required, which helped to extend battery life.

Interrogation of the patient's pacemaker between 1-13-21 and 5-17-21 revealed a four-month progressive increase in premature ventricular contraction (PVC) burden from 21% to 31% despite guideline-directed medical therapy for heart failure with low ejection fraction (see above). Given the patient's infarct history, reentrant ventricular depolarizations from scar tissue were a probable cause of the increase in PVCs.

**Figure 1:**
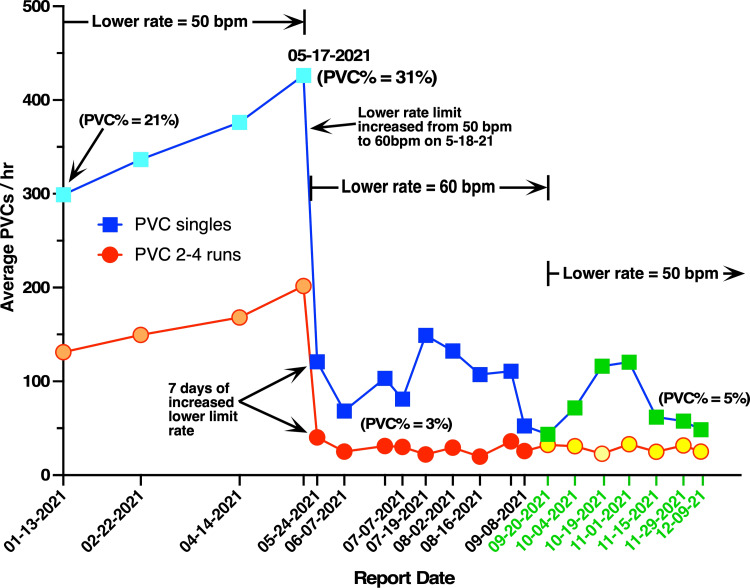
A three-month pacing elevation produced a sustained PVC decrease that persisted even after return to the original heart rate. Raising the pacemaker lower pacing limit from 50 bpm to 60 bpm (beginning on 5-18-2021) decreased the average number of PVCs per hour (ordinate), recorded as both single PVC's (blue) and PVC runs (red). The initial drop in PVC activity from 31% to 3.6% occurred within seven days. Each point represents PVC activity averaged over two-month intervals (prior to 5-17-21) and then averaged over two-week intervals (after 5-24-21). Resetting the lower limit from 60 to 50 bpm after 9-20-2021 did not significantly increase either single PVCs (green) or PVC runs (yellow) for the next three months. The PVC% in parentheses indicate PVCs (single & multiples) as a percentage of total beats. PVC=premature ventricular contraction, bpm=beats per minute

Since a PVC burden of 31% would impair cardiac resynchronization, we investigated whether raising the lower pacing limit from 50 bpm to 60 bpm would reduce PVC levels. The new setting (beginning on 5-18-2021) guaranteed that the patient's HR would always remain equal to or greater than 60 bpm. This resulted in a dramatic drop in PVC activity from 31% to 3.6% within seven days (5-24-21), followed by a further reduction to 3% during the next two weeks (Figure 1). An important consequence of this reduced PVC activity was an improvement in ventricular synchrony from 80% (prior to 5-17-2021) to 95% (on 5-24-2021) with an associated increase in ejection fraction from 37% to 40% (TTE, 2D, M-mode, color and spectral doppler with contrast). This allowed the patient to play six hours/week of strenuous tennis and effectively reclassified him as NYHA class I, high functioning heart failure.

**Figure 2:**
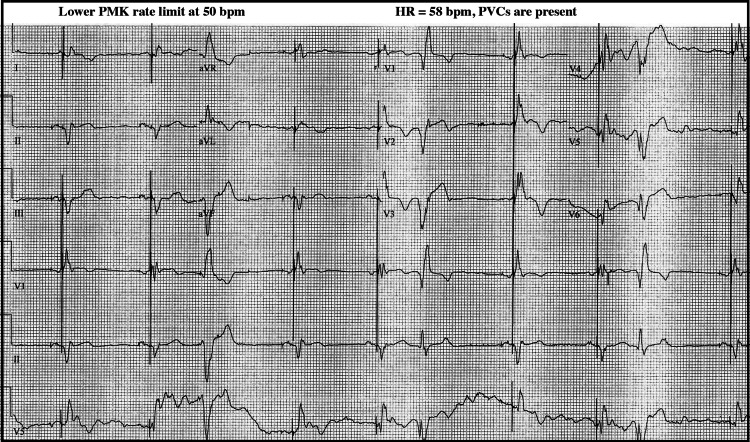
Patient's ECG showing presence of PVCs at the original lower pacing rate limit of 50 bpm. PVC=premature ventricular contraction, HR=heart rate, PMK=pacemaker, bpm=beats per minute

**Figure 3:**
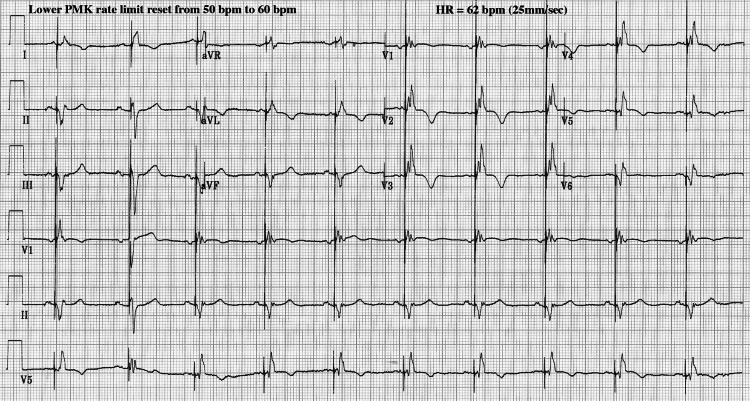
Patient's ECG showing absence of PVCs after elevation of the lower pacing rate limit from 50 to 60 bpm. PVC=premature ventricular contraction, HR=heart rate, PMK=pacemaker, bpm=beats per minute

Prior to 5-17-21, each point in Figure 1 represents PVC activity averaged over two-month intervals. After 5-24-21 each point represents PVC activity averaged over two-week intervals. Percent PVC values were calculated from downloaded pacemaker data, where PVC% = single PVCs/hour plus 3 x PVC runs/hour, divided by average HR (in bpm), divided by 60, assuming a PVC "run" averages three PVCs. Representative ECG records are shown in Figure 2 and Figure 3 for two different pacemaker lower limit settings.

**Figure 4:**
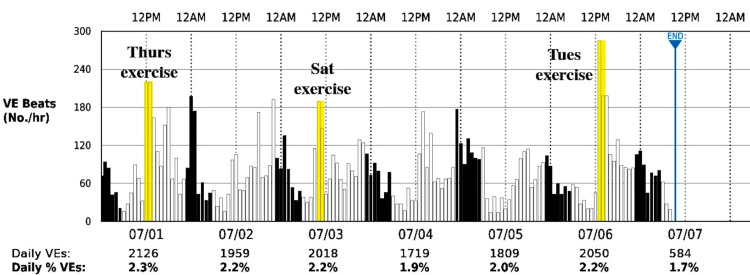
Hourly PVCs recorded continuously during a week of normal activity. Patient records of hourly PVCs (ordinate) were recorded with a Zio XT wearable monitor during periods of normal activity and two-hour periods of strenuous tennis (highlighted in yellow). The average single PVCs/hour for this six day period was 81/hour or 2.4% of total beats. The elevated lower pacing limit of 60 bpm was maintained throughout this period. PVC=premature ventricular contraction, bpm=beats per minute

Since the initial decrease in PVCs (5-24-2021, Figure 1) after lower limit rate elevation could be explained by overdrive pacing [8-10], we decided to look at the direct effect of sudden increases in HR on PVC activity. For two weeks the patient wore a Zio monitor (iRhythm Technologies, Lincolnshire, IL, USA) that recorded PVC activity continuously during rest as well as during strenuous exercise (two hours of tennis three times per week). As indicated in Figure 4, the average weekly PVC burden was between 1.7% and 2.3%, which was similar to global averages recorded by the patient's pacemaker after 5-24-2021 (Figure 1). However, the Zio monitor also indicated that two hours of strenuous tennis (three times per week) approximately doubled the PVC frequency (yellow bars, Figure 4) during a period when HR approached 140 bpm. This suggests a direct correlation between increased HR and increased PVC activity, which argues against simple overdrive suppression of PVCs [8-10]. However, exercise itself (independent of HR) may have contributed to the increase in PVCs, so overdrive suppression might still be important for the initial reduction in PVC on 5-24-2021.

We also investigated whether the long-term pacing-induced drop in PVCs was reversible when the pacemaker lower limit setting was returned to 50 bpm. To our surprise, resetting the lower pacing limit from 60 to 50 bpm did not return PVC levels to their original high level of 31%, but maintained the PVC burden at about 5% (green squares and yellow circles, Figure 1). This persistence of low PVC activity when the lower limit was returned to 50 bpm (9-20-2021 to 12-9-21) argues against simple overdrive suppression of PVCs [8-10].

**Figure 5:**
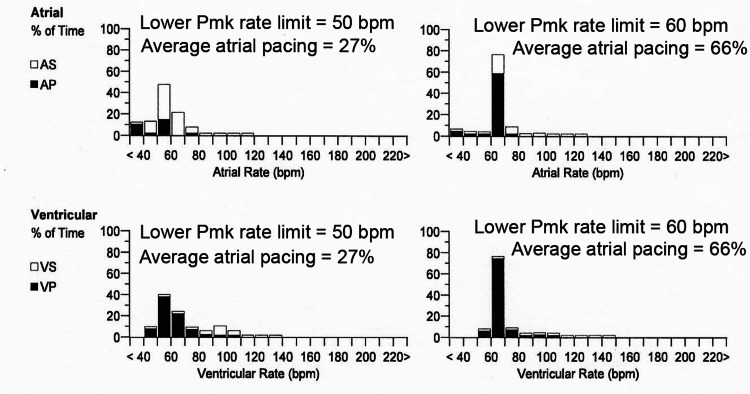
Confirmation that increasing the pacing rate increased atrial pacing despite nebivolol reduction of HR. Elevating the lower rate pacemaker setting from 50 bpm (left) to 60 bpm (right) increased both the percent atrial pacing and the peak (paced and sensed) heart rate by 10 bpm, for this patient whose normal sinus rhythm averaged 49±2 bpm with 10 mg nebivolol (q.d.). Pmk=pacemaker, AS=atrial sensing, AP=atrial pacing, VS=ventricular sensing, VP=ventricular pacing, bpm=beats per minute. Bar graphs show an approximate normal distribution around a peak value of mean heart rate.

To confirm that the pacemaker lower limit adjustment reliably modified average HR we compared atrial and ventricular rates at a lower limit setting of 50 bpm (left side of Figure 5) to atrial and ventricular rates at a lower limit setting of 60 bpm (right side of Figure 5). Since the patient's normal sinus rhythm averaged 49±2 bpm with daily 10 mg nebivolol, it was not surprising that atrial pacing was required only 27% of the time (left side of Figure 5) when the lower limit setting was 50 bpm (prior to 5-17-2021, Figure 1). On the other hand, when the lower limit setting was raised to 60 bpm (5-24-2021 to 9-8-2021), atrial pacing was required 66% of the time to maintain an HR ≥ 60 bpm at the same dose of nebivolol (right side of Figure 5).

## Discussion

The present case describes a dramatic initial decrease in PVCs following elevation of the lower limit rate setting in a patient's implanted pacemaker. This is analogous to the temporary exogenous overdrive pacing sometimes used to treat malignant dysrhythmias that are refractory to cardioversion. However, we also observed the somewhat surprising persistence of low PVC activity even after the pacemaker lower limit was returned to its initial value (50 bpm), close to the patient's original sinus rhythm. We hypothesize that this could have resulted from favorable ventricular remodeling caused by three months of suppressed PVC activity.

This case is important because it revisits an underappreciated, noninvasive technique for PVC reduction in cardiac patients whose PVC burdens lie between 15% and 30% and who may be poor candidates for cardiac ablation because of comorbidities or who are at risk for ischemic stroke. Furthermore, reducing PVC levels with lower limit rate elevation also improves the degree of cardiac resynchronization in LBBB [2,11,12] and reduces cardiomyopathy in patients with structural heart damage [3,6].

Pacing rate adjustment as a strategy for PVC reduction is nothing new [8,13,14]. In hypothermic dogs, ectopic beats occurred more frequently at 50 bpm but decreased as HR was raised, with complete abolition at 75 bpm pacing [15]. Premature ectopic beats were also found more frequently in patients with low HRs arising from complete A-V block [16]. However, clinical pacing adjustments would only be useful for a subset of cardiac patients (about 24%) exhibiting bradycardia-associated PVC activity. Three general subgroups of HR-dependent PVCs have been identified: (1) a bradycardia-enhanced pattern, (2) a tachycardia-enhanced pattern, and (3) an indifferent pattern [17-19]. The tachycardia-enhanced PVC subgroup (28%) can often be helped by beta-blockers, but the bradycardia-enhanced PVC subgroup and the indifferent subgroup (48%) are less amenable to pharmacological therapy. Fortunately, our patient fell into the (slow rate) bradycardia-enhanced subgroup which allowed reduction of his PVC burden from 31% to less than 5% by a simple elevation of his pacing lower limit from 50 bpm to 60 bpm.

Although the mechanism linking slow HR to increased PVC activity is not well understood, it's possible that low HR increases the range of ventricular refractory periods and decreases the fibrillation threshold in the ventricle [15]. Artificial pacing rates of 10 to 20 bpm above sinus rhythm (but slower than tachycardia) have been used to control ventricular arrhythmia without causing an increase in myocardial oxygen demand [20]. In general, the longer the diastolic period the more opportunity for ectopic rhythms. Therefore, in a subset of cardiac patients, where bradycardia exacerbates arrhythmia, rate acceleration can decrease PVC frequency by making refractoriness more uniform, thereby reducing the likelihood of re-entry. This is similar to dynamic overdrive pacing that has been used for short-term suppression of ventricular ectopic activity [14].

From a clinical standpoint, reduction of PVC levels by pacemaker rate elevation would first require assigning patients to one of three HR groups: slow-PVC, fast-PVC, or indifferent; based on short-term ECG recordings during pacing increments from 50 bpm to 70 bpm. Only patients in the slow (bradycardia)-PVC group would safely benefit from increases to their lower limit pacing rate. Patients in the fast-PVC or the indifferent group could not be managed by pacemaker rate adjustments since faster pacing would only increase their PVC burden.

In addition to the observation that chronically increased pacing (50 to 60 bpm) reduced average PVCs/hour from 31% to 3% (Figure 1), we also report the novel finding that PVC levels were maintained below 5% even after the pacemaker's lower limit was returned to its original value of 50 bpm (9-20-2021 to 12-09-2021, Figure 1). This irreversible suppression of PVC activity following a three-month pacing elevation has not been previously reported or described in detail and constitutes a new clinical observation. Unfortunately, we do not understand the cellular mechanism of this phenomenon. One possibility is that the three-month period (5-24-21 to 9-8-2021, Figure 1) of elevated pacing and reduced PVC levels modified an ectopic focus that was responsible for the original PVC level of 31%. Further evaluation of this ventricular remodeling hypothesis would require 3D electrophysiology mapping of the patient's ventricle. However, this type of invasive procedure would not be medically justified except in preparation for cardiac ablation.

Learning points/take-home messages

(1) A simple pacing adjustment can reduce PVCs in cardiac patients with implanted pacemakers. This is an underappreciated, noninvasive option that can diminish PVC burden in a subset of patients having bradycardia-induced ectopic beats, regardless of whether bradycardia is idiopathic or medication related.

(2) For patients with PVCs comprising 10% to 30% of total beats, resetting the pacemaker lower limit from 50 bpm to 60 bpm or 70 bpm can dramatically reduce PVC levels in patients who may be poor candidates for cardiac ablation because of comorbidities or who are at risk for ischemic stroke.

(3) PVC reduction by pacemaker lower limit elevation enhances cardiac resynchronization in LBBB [2,11,12] and reduces cardiomyopathy in patients with structural heart damage [3,6].

(4) This case also highlights the novel finding that three months of accelerated pacing continued to suppress PVC activity even after the lower pacing limit was returned close to the patient's original HR. The cellular mechanism for this persistence of low PVC activity may involve favorable ventricular remodeling.

## Conclusions

This case report highlights the use of a simple pacing adjustment to reduce the PVC burden in cardiac patients with implanted pacemakers. As such, this method is a noninvasive option that can reduce PVC frequency in a subset of patients having bradycardia-induced ectopic beats, regardless of whether the bradycardia is idiopathic or medication related. For patients having PVC burdens between 10% to 30%, elevating the pacemaker lower limit from 50 bpm to 60 bpm or 70 bpm can dramatically reduce PVC levels without the risk and expense of catheter ablation. This quick and simple method of PVC reduction could mitigate cardiomyopathy, while not precluding catheter ablation at a later date.

Finally, we report the novel observation that three months of elevated pacing continued to suppress PVC activity even near the patient's original HR. This raises the possibility that pacing-induced reductions in PVC activity may have remodeled the ventricle to depress the original ectopic focus. However, this explanation remains only a hypothesis since we did not have continuous 3D electrical mapping of the ventricle during the 11-month recording period.
